# Assessment of baroreceptor reflex sensitivity in young obese Saudi males at rest and in response to physiological challenges

**DOI:** 10.14814/phy2.14625

**Published:** 2020-11-15

**Authors:** Abdullah N. AlShahrani, Lubna I. Al‐Asoom, Ahmed A. Alsunni, Nabil S. Elbahai, Talay Yar

**Affiliations:** ^1^ Department of Physiology College of Medicine Imam Abdulrahman Bin Faisal University Dammam Saudi Arabia

**Keywords:** autonomic nervous system, baroreceptor sensitivity, Baroreflex, body mass index, deep breathing, heart rate, isometric exercise, isotonic exercise, obesity

## Abstract

Autonomic imbalance in overweight/obese persons could lead to an increased risk of cardiovascular complications including hypertension and arrhythmias. Baroreceptor reflex sensitivity is a sensitive indicator to detect an altered sympathovagal balance in overweight/obese individuals. This study investigated the effects of overweight/obesity on baroreceptor sensitivity in young Saudi males at rest and in response to physiological challenges.

**Subjects and methods:**

In this cross‐sectional study, spontaneous baroreceptor sensitivity at rest and in response to deep breathing, isometric hand grip exercise and moderate intensity isotonic exercise were recorded in 20 normal weight and 20 overweight/obese subjects. Finger arterial blood pressure signal, recorded through Finometer, was used to calculate baroreceptor sensitivity through cross‐correlation method. The baroreceptor sensitivity data were log transformed before application of parametric tests.

**Results:**

The spontaneous baroreceptor sensitivity was similar in both groups at baseline, but exhibited a significant increase during deep breathing only in normal weight (*p* < .001). Immediately after the isotonic exercise the baroreceptor sensitivity was significantly lower than baseline in both normal weight and overweight/obese and remained significantly lower in overweight/obese individuals compared to normal weight (*p* < .05) throughout the recovery period. There was a significant rise in baroreceptor sensitivity after isometric exercise in overweight/obese group only (*p* = .001). Pearson's correlation showed a significant negative correlation of baroreceptor sensitivity with body mass index during deep breathing (*r* = −.472, *p* = .004) and in post‐isotonic exercise recovery period (*r* = −.414, *p* = .013).

**Conclusion:**

A significantly reduced baroreceptor sensitivity response to deep breathing, reduced baroreceptor sensitivity recovery after isotonic exercise, and an exaggerated shoot up after isometric exercise in overweight/obese suggests an altered sympathovagal balance. Baroreceptor sensitivity measurements in response to physiological challenges, deep breathing, and isotonic exercise, may be more sensitive investigations for detection of early attenuation of cardiac autonomic function. This would enable timely intervention thereby delaying complications and improving the quality of life.

## Graphical abstract

BRS measurements in response to physiological challenges may be more sensitive investigations for the detection of early attenuation of cardiac autonomic function. This would enable timely intervention thereby delaying complications and improving the quality of life.

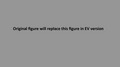

## 1 INTRODUCTION

There is a staggering increase in the prevalence of obesity and its complications throughout the world. According to World Health Organization data, the incidence of obesity has doubled in the last 40 years with about 1.9 billion adults being overweight or obese (WHO, 2018). In Saudi Arabia, the prevalence of obesity in 2014 was estimated to be 24.1% in males and 14% in females with the highest incidence of obesity in the eastern region of the country (Al‐Hazzaa et al., 2014).

Several reports have confirmed the association between obesity and cardiovascular risk in adults as well as in children (Lahey and Khan, (2018); Farmer et al., 2019; Francesquet et al., 2019). The risk of cardiovascular disease in overweight/obese individuals has been linked to impairment of the autonomic nervous system (ANS), characterized by sympathetic overactivity and/or reduction in parasympathetic activity (Guarino et al., 2017). Latent autonomic neuropathy may be present in otherwise healthy overweight/obese individuals. Early detection of cardiac autonomic dysfunction is of major clinical interest, which could lead to a more intensive supervision of overweight/obese and diabetic patients ( Spallone, 2019; Wulsin et al., 2015).

Resting heart rate (HR) is the simplest indicator of cardiovascular health. A high resting HR in overweight/obese individuals has been reported in a number of studies throughout the world including Saudi Arabia (Lee et al., 2014; Yar, 2010). This higher resting HR could reflect an imbalance in sympathovagal control of the heart. Autonomic imbalance predisposes overweight/obese individuals to cardiac arrhythmias, hypertension, and other cardiovascular complications (Lee et al., 2014; Rossi et al., 2015).

Arterial blood pressure (BP) is maintained on a short‐term basis with the help of the baroreceptor reflex involving the vagal and sympathetic systems. The activity of the sympathetic and parasympathetic nervous systems can be monitored in humans by measurements of heart rate variability (HRV) and baroreceptor reflex sensitivity (BRS) (Novak, 2011; Weimer, 2010). Compared to conventional autonomic function tests, the measurement of HRV and BRS provides a better and more sensitive means for early detection of autonomic complications and in prognosis of diseases (Pinna et al., 2015; Spallone, 2019). Baroreceptor function expressed as BRS reflects the integrated capacity of autonomic system, and BRS measurement is considered a powerful and sensitive tool for the assessment of autonomic dysfunction, with useful applications in clinical practice (Pinna et al., 2015). BRS refers to the amount and speed of change in the length of the cardiac period (the inter‐beat interval), in response to and coinciding with change in BP. Systolic BP ramps (increases or decreases) drive inter‐beat interval (IBI) to change through the mediation of baroreceptor reflex. If BRS is reduced, cardiac cycle duration changes less in response to changing BP.

Cardiovascular autonomic reflex tests use provocative physiological maneuvers and measure the heart rate and blood pressure response (Novak, 2011; Spallone et al., 2011). Heart rate response to deep breathing is an easy to perform, sensitive, established test for clinical autonomic testing. The heart rate variation during inspiration and expiration is maximally exaggerated during deep breathing at 6 breaths per minute and this is also associated with variations in the blood pressure (Novak, 2011; Sin et al., 2010). This increase in BP variations over the respiratory cycle, together with the reflex response via arterial baroreflex leads to the increase in cardiac vagal modulation during deep breathing (Stankovski et al., 2013). A physiological increase in BRS with deep breathing has been demonstrated, though the exact underlying mechanism is not clear (Bernardi et al., 2001; Friedrich et al., 2010).

Exercise is a common physiological stress that can help in testing the reserve of a system. Both isometric (static) and isotonic (dynamic) exercise can be applied as a stress to the cardiovascular system (Fletcher et al., 1990). The observed pattern of changes in HR and BP in response to stressors is regulated by the ANS and can be measured (Novak, 2011). It has been suggested that overweight/obese individuals display altered reactions to stressors such as physical exercise (Piccirillo et al., 1996). Isotonic and isometric exercises involve the withdrawal of parasympathetic nervous system activity and activation of the sympathetic system thereby causing a change in BRS, but the extent and mechanism of change could be different in different types of exercises (Weippert et al.,). Both isotonic and isometric exercises have been shown to suppress the BRS and it has also been observed that there is an increase in the BRS in the recovery period after isometric exercise (Taylor et al., 2017; Teixeira et al., 2018).

Most of the investigations dealing with BRS have been performed on non‐Arab subjects and ethnicity has been reported to influence the cardiovascular autonomic responses including BRS and vascular reactivity to exercise (Carnevali et al., 2019; Foulds et al., 2015). There are hardly any studies measuring the BRS in young individuals in Saudi Arabia. Furthermore, no studies are available in Saudi Arabia addressing the effects of obesity or exercise on BRS in young individuals. Therefore, we decided to (i) investigate the effect of obesity on spontaneous BRS in young Saudi individuals at rest and (ii) compare the effects of deep breathing and isometric and isotonic exercises on BRS in normal weight and overweight/obese individuals.

We hypothesized that the resting spontaneous cardiovagal baroreceptor sensitivity will be less in young overweight/obese from that in normal weight individuals. We also hypothesized that the baroreceptor response to physiological challenges would be blunted in overweight/obese compared to normal weight individuals.

## 2 SUBJECTS AND METHODS

The study was performed in the Physiology laboratory, College of Medicine, Imam Abdulrahman Bin Faisal University, Kingdom of Saudi Arabia from September 2015 to March 2016. Approval of research and ethics committee was obtained before starting the project. This study was a cross‐sectional study using convenience sampling to select young (18 to 23 years old) healthy Saudi male university students of Arab ethnicity by advertisement in the university. A sample size of 20 in each group was decided to provide a normal distribution. All the subjects were university students who had undergone a detailed medical checkup in the University clinic. Subjects were screened through history, physical examination and laboratory tests for glucose, lipids, and anemia. Individuals were excluded if they had any systemic disease including diabetes, heart problems, taking any medications that could affect autonomic activity, or any bone or joint problems hindering in exercise. Written informed consent was taken from all the volunteers. All procedures conformed to Declaration of Helsinki.

Each subject visited the laboratory twice. During the first visit to the physiology laboratory, the participants were briefed about the protocol. Height and weight were measured and body mass index (BMI) was calculated. Subjects were divided into two groups based on BMI: normal weight (BMI = 18.5 to 24.9 kg/m^2^) and overweight/obese (BMI ≥25 kg/m^2^). Waist circumference was measured with an inelastic tape to obtain waist‐to‐stature ratio. Predicted maximal heart rate (HR_max_) was calculated with the help of the formula: HR_max_ = 220‐age. Next, an estimation of the workload required to achieve 40% of heart rate reserve was performed through a step‐up protocol of exercise (Garber et al., 2011) on an electronically controlled ergometer bicycle (Monark‐839E). The subject started with 20 watts, and the load was increased by 20 watts at 1‐min intervals till the subject achieved a plateau in HR. HR was continuously monitored through HR display on the computer derived from a T34 Polar chest belt. The calculation of target heart rate at 40% of heart rate reserve was done through Karvonen formula: heart rate reserve = HR_rest_+0.4(HR_max_−HR_rest_).

After an interval of at least 2 days, the subjects visited the laboratory for the second time and were hooked up with the Finometer Pro® (FMS, The Netherlands) for continuous measurements of finger arterial BP, and with the Powerlab® (8/35) system (ADInstruments, Australia) for electrocardiogram and respiratory rate through bioamplifier and the respiratory belt (ADInstruments, Australia), respectively. The data were sampled at a frequency of 1,000 Hz. Resting baseline readings were obtained for 25 min. The HR and BP were measured with a SPOT vital sign® monitor (NY‐13153) during the resting period and during recovery form isotonic exercise. Each subject first performed deep breathing at a rate of six breaths/min for one minute. The subjects were trained to perform the gradual deep inhalation for 5 s and gradual exhalation for 5 s. They were encouraged not to take a sudden breath or hold the breath. Maximal voluntary contraction of the dominant hand was measured by having the subject perform isometric hand grip exercise with a dynamometer (ML T003/D, AD Instruments, Australia) three times and the maximum value was selected. Then each subject performed isometric handgrip exercise at 30% of maximum voluntary contraction for 3 min (Hilz & Dütsch, 2006). During isometric exercise, the subjects had visual feedback from the LabChart computer screen to maintain force output to the predetermined percentage of maximum contraction. The subjects were encouraged not to tense any other muscles apart from those of the forearm used in the handgrip. In addition, they were asked to maintain the squeeze on the dynamometer for the full duration of the exercise period (3min). Monitoring of respiratory movements ensured that Valsalva maneuver was not performed inadvertently. This was followed by moderate intensity isotonic exercise at 40% of heart rate reserve on an electronically controlled ergometer bicycle (Monark‐839E) for 20 min. After cessation of isotonic exercise, the recording was continued for 30 min. Just before the end of recording session the deep breathing maneuver was repeated.

Baroreceptor reflex sensitivity was calculated offline with the help of dedicated program (PRVBRS) provided by FMS (The Netherlands). The program uses cross‐correlation method to calculate the BRS (Westerhof et al., 2004). It computes the correlation between beat to beat systolic blood pressure and inter‐beat interval in a sliding 10‐s window, with delays of 0 to 5 s for interval. The delay with the greatest significant positive correlation is selected and the slope and the delay are recorded as one BRS value. BRS readings were averaged over at least 2–5 min except in deep breathing where the maneuver itself was for 1 min only. The measurement of BRS by cross‐correlation method has been tested and validated and is able to provide a greater number of instantaneous values of BRS in a given time compared to sequence method of BRS (Wesseling et al., 2017; Westerhof et al., 2004). The inbuilt return‐to‐flow and height correction facilities have improved the accuracy and reliability of Finometer device that is clinically acceptable (Guelen et al., 2008).

Statistical analysis was performed using the Statistical Package of Social Science (SPSS) version 26 (IBM, USA). Descriptive statistics were used to calculate means and standard deviation for all the variables. All the subjects served as their own control to determine the difference between pre and postmaneuver effects. Shapiro–Wilk test was used to test the normality of data. The skewed data of BRS were natural log transformed for further analysis by parametric tests. A comparison between the groups was performed to determine the differences between the normal weight and overweight/obese individuals. A paired *t* test was used to compare the significance of difference between resting and postexercise HR, BP, and BRS in each subject. An unpaired *t* test was used to measure the significance between groups. A “*p*” value of < .05 was taken as statistically significant.

Data from one normal weight and one overweight/obese subject were excluded because of erroneous recordings.

## 3 RESULTS

Obesity indices of the normal weight and overweight/obese groups are presented in Table 1. The two groups were significantly different in their obesity indices BMI, waist circumference, and waist‐to‐stature ratio. Age and height of the subjects were not significantly different. Resting cardiovascular parameters—HR, systolic BP (SBP), diastolic BP (DBP), pulse pressure (PP), and mean arterial BP (MABP) were higher in the overweight/obese group compared to normal weight group, but the difference was not statistically significant (Table 1).

**Table 1:** Comparison of baseline characteristics (the obesity indices, resting cardiovascular parameters, respiratory rate) between the normal weight and overweight/obese groups

	Normal weight (*n* = 19)	Overweight/obese (*n* = 19)	P value
Age (years)	21.05 ± 1.93	21.0 ± 1.62	0.93
Weight (Kgs)	65.15 ± 9.21	98.71 ± 20.48	0.000*
Height (meters)	1.70 ± 0.05	1.70 ± 0.07	0.705
BMI (Kg/m^2^)	22.6 ± 2.81	33.8 ± 5.59	0.000*
Waist circumference (cm)	79.7 ± 9.48	105 ± 12.8	0.000*
Waist‐to‐stature ratio	46.94 ± 5.55	61.8 ± 6.74	0.000*
Resting HR (bpm)	74 ± 9.7	79.3 ± 14.28	0.14
Resting SBP (mm Hg)	109.9 ± 7.08	115 ± 10.8	0.11
Resting DBP (mm Hg)	68.79 ± 7.29	71 ± 7	0.26
Resting PP (mm Hg)	41 ± 9.2	43 ± 8.3	0.45
Resting MABP (mm Hg)	82.5 ± 5.78	85.8 ± 7.51	0.13
Resting respiratory rate (per minute)	19.2 ± 2.3	22.2 ± 5.84	0.097

Abbreviations: BMI, Body mass index, HR, heart rate, SBP, systolic blood pressure, DBP, diastolic blood pressure, PP, pulse pressure, MABP, mean arterial blood pressure.

Data are in mean ± standard deviation.

* *p* < .01 using independent samples *t* test.

**Figure 1:**
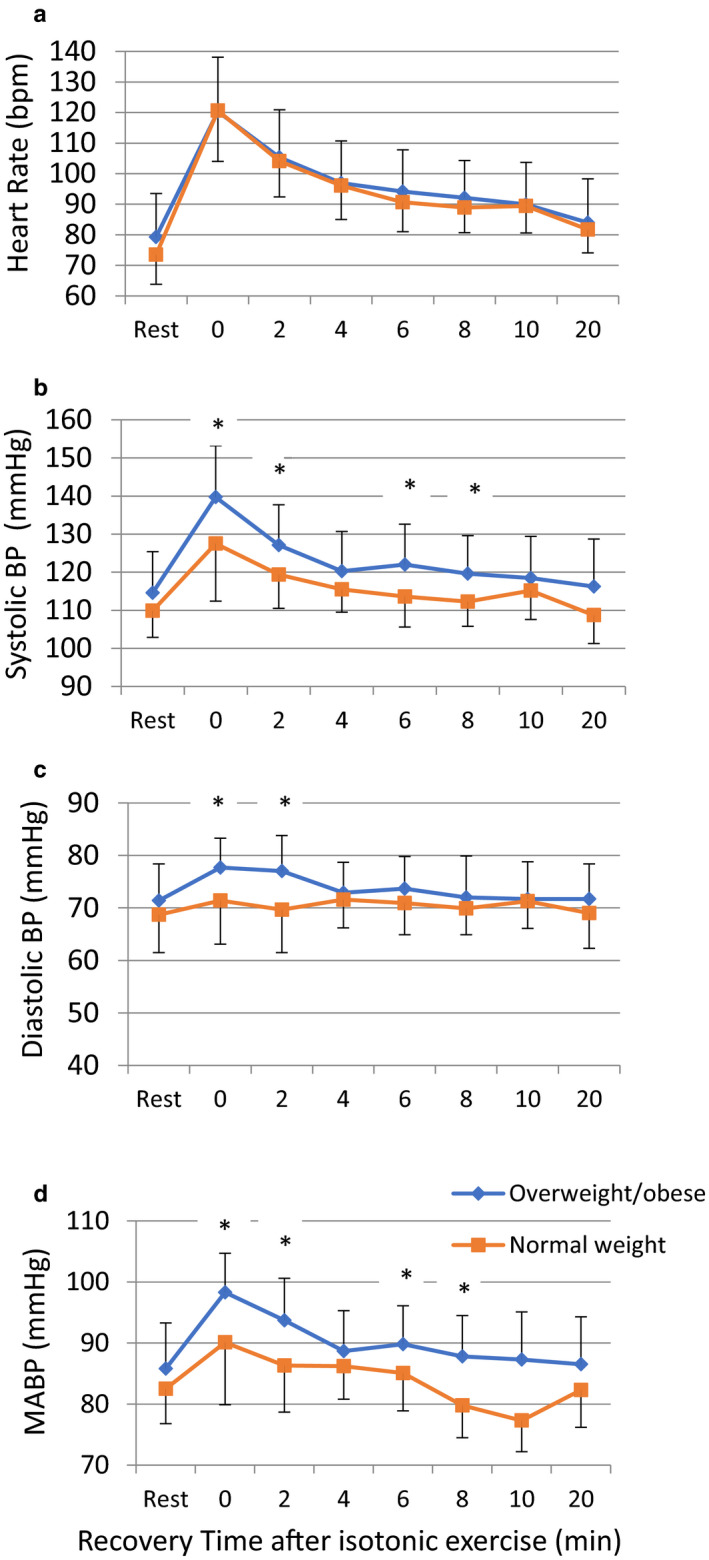
Comparison of (a) heart rate, (b) systolic blood pressure, (c) diastolic blood pressure, (d) mean arterial blood pressure at rest and during recovery after moderate intensity continuous exercise, between obese and normal weight subjects. HR: Heart Rate; SBP: Systolic Blood Pressure, DBP: Diastolic Blood Pressure, MABP: Mean arterial blood pressure; bpm: beats per minute. * *p* < .01 using independent samples *t* test

Comparison of hemodynamic parameters, that is, HR, SBP, DBP, PP, and MABP at rest and during various points in recovery after isotonic constant exercise revealed no significant difference in HR between normal weight and overweight/obese groups. However, SBP immediately after cessation of the isotonic exercise and at 2,6,8 min postexercise was significantly higher in overweight/obese compared to normal weight. DBP was significantly higher in overweight/obese compared to normal weight at 0‐ and 2‐min postisotonic exercise. MABP was significantly higher at 0, 2, 6, and 8 min postisotonic exercise in overweight/obese compared to normal weight (Figure 1). Blood pressure differences in the recovery period became insignificant thereafter. Comparing the hemodynamic parameters at rest and postisometric handgrip exercise yielded no significant difference between overweight/obese and normal weight, (Data are not shown).

The respiratory rate was slightly higher in obese compared to normal weight both at baseline (mean ± *SD*: overweight/obese:22.2 ± 5.84 per min; normal weight: 20.1 ± 1.75;) and at 30 min after isotonic exercise (22.8 ± 5.62; 19.2 ± 2.3), but the difference was nonsignificant (*p* = .097; *p* = .106, respectively). The respiratory rate at 30 min after isotonic exercise had recovered to near baseline in both the normal weight (*p* = .152) and the overweight/obese (*p* = .255) groups.

The baseline resting BRS was 10.60 ± 4.35 ms/mm Hg in the whole cohort of 38 subjects with slightly higher values in the normal weight group compared to the overweight/obese group (normal weight: 11.05 ± 4.20; overweight/obese: 9.80 ± 4.56 ms/mm Hg). During deep breathing the BRS increased to 16.37 ± 5.61 in normal weight (*ca*. 48%) and to 10.31 ± 4.87 ms/mm Hg in overweight/obese (*ca*. 5%). Similarly in the postisotonic exercise period at 25 min, deep breathing led to an increase in BRS of ca. 38% (from 9.68 ± 3.12ms/mm Hg to 11.08 ± 3.99ms/mm Hg) in normal weight compared to a slight decrease (−8%) in BRS in overweight/obese (from 6.39 ± 3.65ms/mm Hg to 5.87 ± 3.78ms/mm Hg).

**Figure 2:**
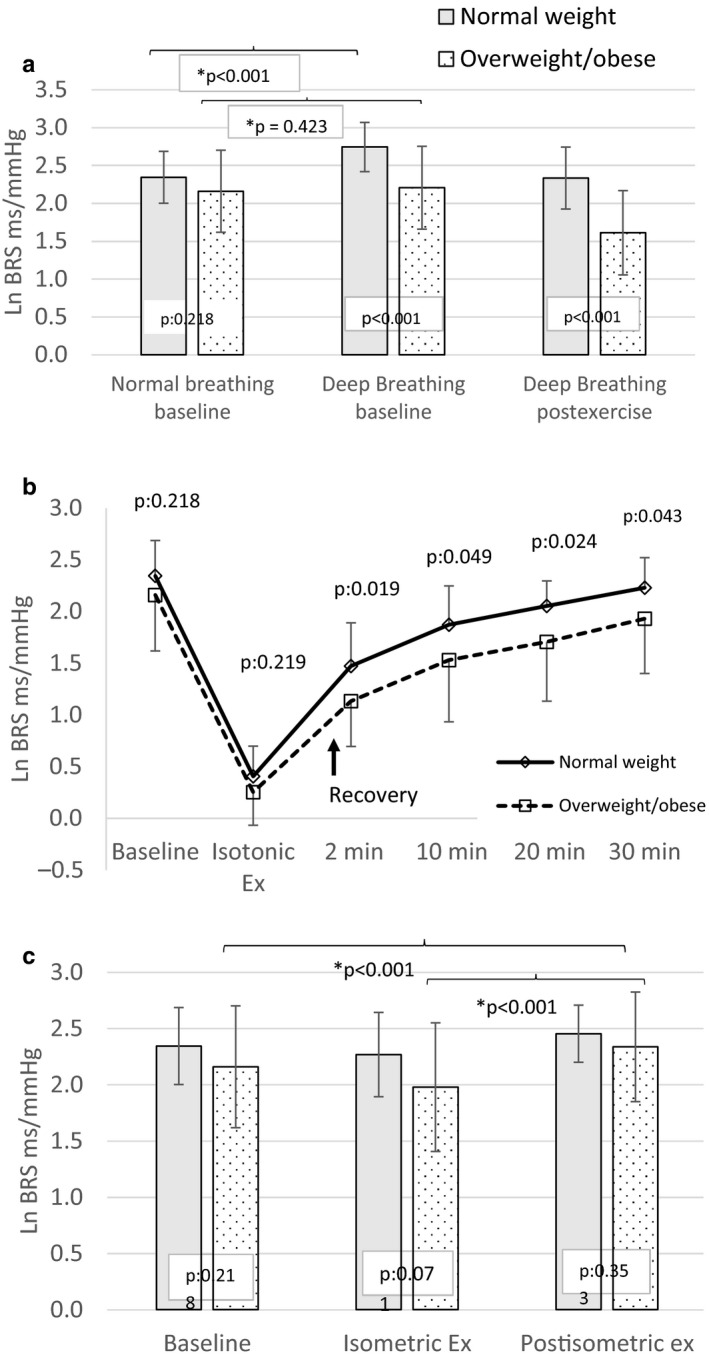
Comparison of ln BRS between normal weight (*n* = 19) & overweight/obese (*n* = 19) subjects at rest, in response to Physiological challenges. Data are presented as means ± standard deviations. Students' unpaired *t* test done for comparison between normal weight and overweight/obese groups. Within group comparisons with paired *t* tests. Ln BRS: natural logarithm of baroreceptor sensitivity. (a) Normal breathing at rest, during deep breathing at baseline, and during deep breathing at 25 min during recovery after isotonic exercise. (b) At rest, during after isotonic, during recovery after isotonic exercise. (c) At rest, during isometric handgrip exercise at 30% of maximum voluntary force, and after isometric exercise

Figure 2a shows that ln BRS at baseline during normal breathing was less in overweight/obese compared to normal weight, but the difference was not significant. Compared to normal breathing there was a significant increase in ln BRS during deep breathing (*p* < .001) in normal weight only whereas in the overweight/obese the increase in BRS was nonsignificant (*p* = .423). This significant increase in ln BRS in normal weight during deep breathing led to the manifestation of a significant difference (*p* < .001) in ln BRS between normal weight and overweight/obese during the deep breathing. In the deep breathing performed during the postisotonic exercise recovery period at the end, the BRS was significantly less in the overweight/obese compared to normal weight (*p* < .001).

Figure 2b shows the ln BRS values at rest, during exercise, and postisotonic constant exercise recovery at the intervals (2, 10, 20 & 30 min) in normal weight and overweight/obese. The overweight/obese subjects had lower ln BRS values at rest compared to normal weight, but the difference was not significant (*p* = .218). During the constant load isotonic exercise at 40% of VO_2max_, the BRS was drastically reduced (*p* < .001) in both groups (normal weight = 1.56 ± 0.48 ms/mm Hg, overweight/obese = 1.35 ± 0.43), but the difference between the groups was not significant (*p* = .244). Postexercise recovery of BRS in overweight/obese was delayed compared to normal weight and BRS value remained significantly lower in overweight/obese till 30 min of postexercise recording compared to normal weight (*p* = .043). The BRS recovered fully in normal weight at 30 min (*p* = .248) compared to the initial resting baseline, but it remained significantly lower (*p* = .012) compared to baseline rest in the overweight/obese group.

Figure 2c shows the comparison of ln BRS values at rest, during isometric exercise and postisometric exercise depicting a nonsignificantly lower ln BRS at rest (*p* = .218), during (*p* = .071) and postisometric exercise (*p* = .353) in overweight/obese compared to normal weight. The ln BRS value after isometric exercise in overweight/obese was nonsignificantly lower (1.98 ms/mm Hg) compared to normal weight group (2.27 ms/mm Hg) (*p* = .071). Within the groups’ comparison showed higher postisometric exercise ln BRS compared to resting ln BRS in normal weight (resting:2.34ms/mm Hg, Postisometric exercise:2.45), but the difference was not significant (*p* = .155). In contrast, the overweight/obese group exhibited significantly higher postisometric exercise ln BRS (2.34ms/mm Hg, *p* = .006) compared to resting (2.16ms/mm Hg) as well as compared to ln BRS during isometric exercise (1.98 ms/mm Hg; *p* < .001).

**Figure 3:**
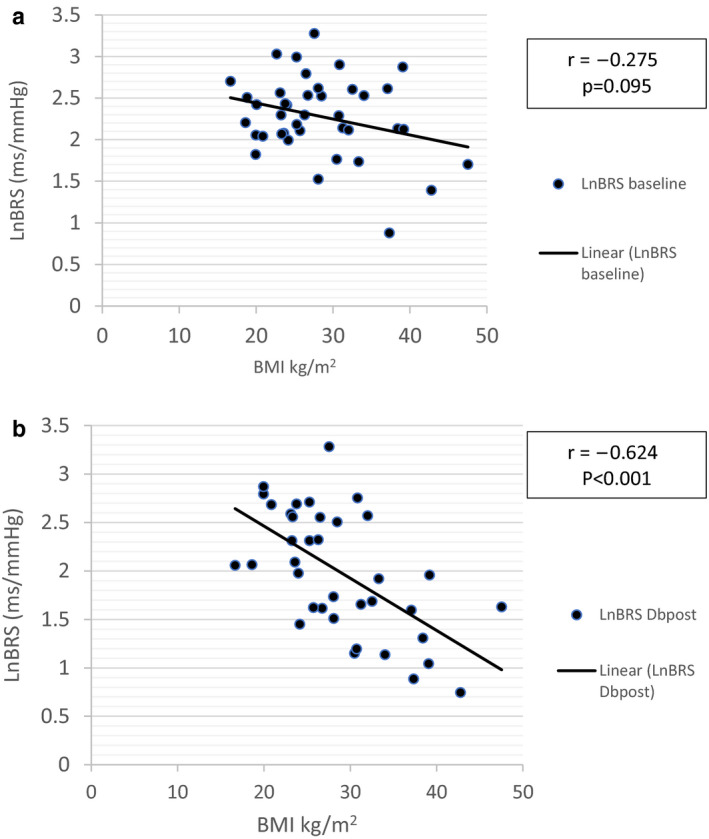
Pearson's correlation of BMI with lnBRS in whole cohort of subjects. (a) During rest at baseline. (b) During deep breathing in the recovery period after isotonic exercise. *n* = 38; Ln BRS: natural logarithm of baroreceptor sensitivity. BMI: body mass index, r: Pearson's correlation coefficient, Dbpost: deep breathing in postisotonic exercise recovery period

Table 2 shows that the obesity indices: BMI, waist circumference, and waist‐to‐stature ratio were negatively correlated with ln BRS at rest (baseline), during isometric exercise and postisometric exercise, but the relationship was not significant. During deep breathing and in recovery period after isotonic exercise, BMI, waist circumference, and waist‐to‐stature ratio were significantly and negatively correlated with ln BRS. Figure 3a shows the nonsignificant negative relationship of BMI with ln BRS at baseline compared to the significant negative relationship during deep breathing after dynamic exercise (Figure 3b).

**Table 2:** Correlation of the natural logarithm of baroreceptor sensitivity with obesity indices

Variables	r with BMI	P value	r with Waist circumference	P value	r with waist‐to‐hip ratio	P value
Ln BRS baseline	−0.275	0.095	−0.298	0.084	−0.219	0.187
Ln BRS during deep breathing at baseline	−0.472	0.004	−0.400	0.016	−0.368	0.027
Ln BRS during isometric exercise	−0.224	0.182	−0.153	0.365	−0.115	0.499
Ln BRS postisometric exercise	−0.284	0.084	−0.313	0.055	−0.246	0.137
Ln BRS during postisotonic exercise	−0.415	0.013	−0.391	0.020	−0.384	0.023
Ln BRS during deep breathing in recovery after postisotonic exercise	−0.624	<0.001	−0.521	<0.001	−0.586	<0.001

Abbreviations: BRS, baroreceptor sensitivity, BMI, body mass index; Ln, natural logarithm; r, Pearson's correlation coefficient.

## 4 DISCUSSION

This study evaluated the resting BRS and effects of physiological challenges on BRS in young normal weight and overweight/obese Saudi males. The resting sitting BRS in young healthy normal weight and overweight/obese males was similar in magnitude to what has been observed in various studies conducted in American and European subjects of comparable age (Bernardi et al., 2011; Iellamo et al., 1996; Kim & Euler, 1997; Westerhof et al., 2006). Baroreceptor sensitivity has been shown to be influenced by many factors such as age, gender, body mass index, physical, and mental stress as well as deep breathing (Kardos et al., 2001; Skrapari et al., 2006). Contrary to these findings we did not observe a significant difference in resting BRS between normal weight and overweight/obese groups of young Saudi males, though the difference became apparent in response to physiological challenges. One probable explanation of this difference in observation could be that the sample size in our study was not large enough. The fact that the difference in BRS became significant in response to physiological challenges further supports this assumption. Another possibility is that the subjects varied in their age and duration and type of obesity as seen in the study by Beske et al, (Beske et al., 2002). In contrast to their study, all of our subjects were young and it is documented that BRS declines with advancing age (Kardos et al., 2001). Autonomic depression, characterized by a reduction in both sympathetic as well as parasympathetic activity is also dependent upon the duration of obesity (Nagai et al., 2003; Williams et al., 2019).

On a similar note, the inverse relationship between spontaneous BRS and BMI as seen in our study has also been described in normal weight and overweight/obese subjects in earlier studies (Beske et al., 2002; Kardos et al., 2001). The negative correlation of lnBRS with BMI in deep breathing reflects a higher sympathetic inhibition and parasympathetic enhancement in normal weight compared to overweight/obese in which the sympathetic inhibition and parasympathetic activation were probably of less magnitude. Thus, the BRS increased in those with normal weight and did not change much in overweight/obese during deep breathing leading to a steeper relationship between ln BRS and BMI (Figure 3b).

The responses to challenges of deep breathing and moderate intensity dynamic (isotonic) exercise were different between the two groups. Deep breathing at 6 breaths/min led to a significant increase in BRS both at baseline (48%) as well as in the postisotonic exercise recovery period (38%) in the normal weight individuals, whereas such an increase in BRS was not exhibited by overweight/obese (5%). A significant rise in BRS during deep breathing (ca.45%) compared to normal breathing was demonstrated by Tank et al which is very similar to what we have observed in normal weight subjects in our study, but quite different from the overweight/obese group in this study (Tank et al., 2000). A similar increase in BRS during deep breathing has been observed in healthy individuals in a number of previous studies (Calcaterra et al., 2013; Joseph et al., 2005; Li et al., 2018; Russo et al., 2017). Our results are in contrast to those of Calceterra et al, who observed a significantly greater increase in BRS in response to deep breathing in overweight/obese group compared to normal weight (Calcaterra et al., 2013). The discrepancy might be explained on the basis of age and gender of the subjects. The subjects in Calceterra study were children and adolescents of both genders as against our subjects who were all adult males. Our subjects thus probably had a longer standing obesity leading to significant greater changes in autonomic balance in form of sympathoexcitation (Rosengård‐Bärlund et al., 2009, 2011; Williams et al., 2019). It has also been shown that sympathoexcitation in response to insulin is exhibited by obese males (Young et al., 2010), but not by obese females (Shi et al., 2019, 2020). Although the precise mechanisms underlying the rise in BRS during deep breathing are not clear, but it is capable of reducing the sympathetic activity and enhancing parasympathetic activity (Eckberg et al., 1985; Li et al., 2018; Seals et al., 1993). Muscle sympathetic nerve activity is reduced maximally at the end of inspiration (Oneda et al., 2010) and the accompanying mechanical enlargement of thoracic cage enhances venous return and right end diastolic volume. As the intra‐pericardium volume remains roughly the same it would cause a reduction in the left end diastolic volume and as a consequence, there would be a decrease in the left stroke volume, cardiac output and blood pressure during the inspiratory phase. Expiration would cause the opposite effect, that is, an increase in blood pressure. Thus, cyclic increment and decrement in BP occur during breathing which can secondarily induce a breathing variability of HR via baroreflex effect (Toska & Eriksen, 1993). Both central and peripheral pathways may be involved in cardiac vagal response to deep breathing (Sin et al., 2010). It is possible that there is reduced chemoreflex sensitivity during deep breathing that leads to reduced sympathetic tone or enhanced vagal modulation (Bernardi & Bianchi, 2016; Stankovski et al., 2013).

The BRS decreased significantly and to the same extent during submaximal dynamic exercise in both groups, but the recovery in 30 min postexercise period was slower and of significantly smaller magnitude in overweight/obese compared to normal weight. In agreement with our study, it has been shown previously that BRS decreases progressively from rest to maximal exercise (Iellamo, 2001; Iellamo et al., 1997). Withdrawal of parasympathetic activity and enhanced sympathetic activity during both the dynamic and the isometric exercise have been shown to reduce BRS (Ogoh et al., 2005).

The recovery of BRS after dynamic exercise was significantly slower and less in magnitude in overweight/obese compared to normal weight as evident from the fact that BRS value was only slightly less at 30min in normal weight compared to baseline resting value, whereas it was significantly lower than the resting in overweight/obese. As the heart rates and respiratory rates had returned to near baseline and were similar in the two groups at around 30 min after isotonic exercise it is unlikely that the difference of BRS between the two groups was either an effect of heart rate or respiratory rate. BRS measures, in general, do not need strict control of respiratory pattern especially at higher respiratory rates (Bernardi et al., 2011). The difference in time course of the short term BRS recovery between normal weight and overweight/obese has not been reported before. BRS values have been reported to be reduced significantly at 30 min after mild to heavy dynamic exercise and to be restored back to the pre‐exercise levels at varying times depending on the severity of exercise (Taylor et al., 2017). It has been observed that BRS decreased in young healthy males in response to 30‐min mild exercise and was restored to initial resting value within 20 min after exercise (Raczak et al., 2005). Light aerobic exercise led to a reduction in BRS that could be observed at 15min postexercise, but full recovery had taken place by 60 min (Reynolds et al., 2017). In contrast the higher intensity exercise led to slower recovery of BRS and it was below baseline at 60 min. An intense graded maximal exercise was followed by increased BRS persisting for 24 hr (Convertino & Adams, 1991).

Another factor that affects the resting BRS and its reactivity is ethnicity. The resting pre‐exercise BRS was significantly less in age and body mass index‐matched indigenous Canadians compared to European participants (Foulds et al., 2015). In addition, the postsubmaximal exercise BRS and SBP were significantly reduced compared to baseline in the Europeans whereas there was a nonsignificant difference in BRS and SBP in the indigenous people. In contrast to submaximal exercise, the maximal exercise led to a highly significant decrease in BRS in both groups (Foulds et al., 2015). Our subjects (both normal weight and overweight/obese group) behaved in a manner similar to the European participants in response to submaximal exercise by exhibiting a reduction in BRS in postexercise period. Given that there are differences in regulation of physiological variables such as heart rate variability, blood pressure, responses to challenges such as exercise and responses to drugs for treatment of certain common ailments such as hypertension (Drew et al., 2020; Foulds et al., 2015; Hill et al., 2015; Hill & Thayer, 2019; Johnson, 2008), it is important to study the basic physiological differences in various ethnicities. Our study is a first step in this direction where we have studied the resting BRS and the effect of physiological challenges on BRS in individuals of Arab ethnicity.

The recovery time depends on intensity, duration and type of exercise and the recovery patterns of HRV and BRS indirectly reflect the return to homeostasis characterized by an initial parasympathetic activation and slower sympathetic withdrawal. The overweight/obese group in our study, having a slower recovery characterized by a reduced BRS level, signify heightened sympathetic activity and/or reduced parasympathetic activity (Grassi et al., 1998; Paula et al., 2019; Reynolds et al., 2017). Moderate intensity exercise led to an increase in sympathetic activity evidenced by an increase of catecholamines that persisted for at least 3 hr after exercise (Halliwill et al., 1996). The second observation of a more pronounced difference in response to deep breathing in the recovery period after dynamic exercise between normal weight and overweight/obese lends further support to this suggestion of a higher sympathetic activity in overweight/obese compared to normal weight. It has also been documented that high plasma norepinephrine concentration during dynamic exercise attenuates parasympathetic control of heart rate ( Miyamoto et al., 2003). Precise alterations in the cardiovascular and hemodynamic parameters under control of sympathovagal balance play a key role in meeting the metabolic demands of the working skeletal muscles during exercise (Fadel & Raven, 2012). The major reflex adjustment that occurs is through the baroreceptors that could be reset during exercise so that the HR does not slow down in response to the higher blood pressure that is required under conditions of exercise (Fadel & Raven, 2012; Perini & Veicsteinas, 2003). The exaggerated dominant sympathetic activity in overweight/obese in our study was also manifested by the higher SBP, DBP, and MABP postisotonic constant exercise compared to normal weight. Stimulation of chemoreceptors by hypoxia or hypercapnia that could be present in the overweight/obese in the postexercise recovery period may reduce BRS through sympathetic activation though we did not measure the oxygen and carbon‐dioxide levels (Bernardi & Bianchi, 2016).

There was a significant increase in BRS after the isometric exercise in overweight/obese compared to the value during isometric handgrip exercise as well as the baseline. The normal weight group also behaved in a similar pattern, but the changes were not significant. A similar overshoot has been observed by Dipla et al (Dipla et al., 2010) in lean and obese boys, but they suggest that the mechanism of alteration of arterial pressure could be different. An exaggerated sympathetic and pressor response to isometric exercise was demonstrated in aged hypertensive individuals compared to normotensive subjects (Delaney et al., 2010). A similar heightened response could be present in our overweight/obese subjects compared to a minimal response by normal weight subjects. Isometric handgrip exercise at 30% of maximal voluntary contraction involves only small muscles of one hand and is not expected to stimulate a widespread sympathetic activation and therefore BRS may be maintained during isometric exercise (Ebert, 1986). The intensity of isometric exercise at the onset of exercise could be the factor that has an influence on the BRS (Fisher et al., 2007). It has been suggested that though the cardiac baroreflex control is dynamically modulated throughout the isometric exercise, the baroreflex blood pressure regulation is well maintained. A slightly higher BRS after exercise is indicative of a quick parasympathetic takeover in both groups and may suggest that cardiovagal activity is similar in both groups, but the observed difference was probably a reflection of a persistent sympathetic activity even at rest (Perini & Veicsteinas, 2003). On the contrary parasympathetic withdrawal has been suggested to be more important than the sympathetic overactivity in obese (Pal et al., 2012). The resting heart rate, systolic, and diastolic blood pressures were higher in the overweight/obese, but the difference was not significant. Finally, the SBP, DBP, and MABP were significantly higher in the overweight/obese during the recovery period after isotonic exercise. A delay in the recovery of the normal, resting autonomic regulation of the heart rate following exercise may indicate subclinical pathology, and a prolonged tachycardia may predict mortality (Brown & Brown, 2007). The raised blood pressure as seen in this study could be due to a reduced BRS and is likely to lead to an increased blood pressure variability which can further aggravate the reduction in BRS (Rosengård‐Bärlund et al., 2011). Baroreflex failure would also lead to orthostatic hypotension and loss of diurnal variation in BP (Schrezenmaier et al., 2007). Obesity is closely associated with hypertension (Foulds et al., 2012). Normotensive adults with a family history of hypertension have been reported to exhibit a reduced BRS (Boutcher et al., 2011). Enhanced sympathetic drive has been observed before diagnosis in prehypertensive individuals who have a high BMI (Egan & Stevens‐Fabry, 2015). Cardiac autonomic neuropathy is now recognized to be present in obesity in addition to being a feature of diabetes (Spallone, 2019; Williams et al., 2019). It is documented that obesity leads to thickening of arteries and loss of elasticity of carotid walls leading to reduced sensitivity of BR (Skrapari et al., 2006). Increased physical activity is associated with lower thickness of intima‐media and improved BRS (Raczak et al., 2006).

### 4.1 Limitations

Although the sample size was modest, but most of the studies regarding the effects of exercise on BRS had similar or even smaller sample size (Foulds et al., 2015; Niemela et al., 2008; Reynolds et al., 2017). The study included only male participants of young age and therefore the results cannot be generalized to females and other age groups, but with small sample size it was necessary to control for these as age and gender are known to affect BRS (Kardos et al., 2001). The recovery period was limited to 30 min though the effects of exercise are documented to linger on for longer periods (Convertino & Adams, 1991). We focused only on cardiovagal BRS and did not investigate the sympathetic arm of BRS which could have provided important information about differences in vascular response in two groups. We also did not measure the plasma catecholamine levels which could have provided a greater insight in differences in sympathetic activation (Halliwill et al., 1996).

## 5 CONCLUSION

To the best of our knowledge, this is the first study to measure BRS in young Saudi individuals of Arab origin. The BRS values determined were comparable to the ones cited in the literature about Europeans and American. Compared to normal weight subjects the BRS was lower in overweight/obese at rest, but the difference became significant only in response to autonomic reactivity tests (physiological challenges). This could be indicative of a latent sympathovagal imbalance in overweight/obese that could be explored by measuring spontaneous BRS and its response to physiological challenges such as deep breathing or exercise for an earlier detection of autonomic dysfunction.

## CONFLICT OF INTEREST

The authors declare no conflict of interest.

## AUTHORS’ CONTRIBUTION

All authors are responsible for the design and conceptualization of the study, subject recruitment, data collection and analysis, manuscript writing, review, and approval.
